# Patterns and Predictors of Engagement With Digital Self-Monitoring During the Maintenance Phase of a Behavioral Weight Loss Program: Quantitative Study

**DOI:** 10.2196/45057

**Published:** 2023-07-18

**Authors:** Nicole Crane, Charlotte Hagerman, Olivia Horgan, Meghan Butryn

**Affiliations:** 1 Center for Weight, Eating, and Lifestyle Science Department of Psychological and Brain Sciences Drexel University Philadelphia, PA United States

**Keywords:** weight loss, digital technology, diet, exercise, behavior change, mobile phone

## Abstract

**Background:**

Long-term self-monitoring (SM) of weight, diet, and exercise is commonly recommended by behavioral weight loss (BWL) treatments. However, sustained SM engagement is notoriously challenging; therefore, more must be learned about patterns of engagement with digital SM tools during weight loss maintenance (WLM). In addition, insight into characteristics that may influence SM engagement could inform tailored approaches for participants at risk for poor adherence.

**Objective:**

This study explored patterns of digital SM of weight, diet, and exercise during WLM (aim 1) and examined timing, patterns, and rates of disengagement and reengagement (aim 2). This study also assessed relationships between individual-level factors (weight-related information avoidance and weight bias internalization) and SM engagement (aim 3).

**Methods:**

Participants were 72 adults enrolled in a BWL program consisting of a 3-month period of weekly treatment designed to induce weight loss (phase I), followed by a 9-month period of less frequent contact to promote WLM (phase II). Participants were prescribed daily digital SM of weight, diet, and exercise. At baseline, self-report measures assessed weight-related information avoidance and weight bias internalization. SM adherence was objectively measured with the days per month that participants tracked weight, diet, and exercise. Repeated-measures ANOVA examined differences in adherence across SM targets. Multilevel modeling examined changes in adherence across phase II. Relationships between individual-level variables and SM adherence were assessed with Pearson correlations, 2-tailed independent samples *t* tests, and multilevel modeling.

**Results:**

During WLM, consistently high rates of SM (≥50% of the days in each month) were observed for 61% (44/72) of the participants for exercise, 40% (29/72) of the participants for weight, and 21% (15/72) of the participants for diet. Adherence for SM of exercise was higher than that for weight or diet (*P*<.001). Adherence decreased over time for all SM targets throughout phase II (*P*<.001), but SM of exercise dropped off later in WLM (mean 10.07, SD 2.83 months) than SM of weight (mean 7.92, SD 3.23 months) or diet (mean 7.58, SD 2.92 months; *P*<.001). Among participants with a period of low SM adherence (ie, <50% of the days in a month), only 33% (17/51 for weight, 19/57 for diet) to 46% (13/28 for exercise) subsequently had ≥1 months with high adherence. High weight-related information avoidance predicted a faster rate of decrease in dietary SM (*P*<.001). Participants with high weight bias internalization had the highest rates of weight SM (*P*=.03).

**Conclusions:**

Participants in BWL programs have low adherence to the recommendation to sustain daily SM during WLM, particularly for SM of diet and weight. Weight-related information avoidance and weight bias internalization may be relevant indicators for SM engagement. Interventions may benefit from innovative strategies that target participants at key moments of risk for disengagement.

## Introduction

### Background

Self-monitoring (SM) of weight, diet, and exercise is a cornerstone of behavioral weight loss (BWL) treatment [1], and daily SM of these key weight control behaviors is associated with better weight loss and maintenance [2-4]. Nevertheless, despite its importance, rates of engagement with SM are modest and tend to decrease over time, particularly during weight loss maintenance (WLM) [5,6]. With the rise of digital devices for SM (eg, food tracking apps, digital scales, and Fitbit activity trackers), technology is routinely being incorporated into BWL programs [7,8]. Digital devices may facilitate SM adherence by decreasing burden via time-saving features (eg, nutrition databases and saving frequent foods), portability for real-time monitoring, and passive recording of behavior (ie, Fitbit wristwatch for active minutes) [7]. Research shows that adherence to SM via digital format is higher than that via traditional methods [9], likely for these reasons, and that digital SM facilitates calorie reduction and weight loss [10].

There is a growing body of literature examining rates of engagement with digital SM formats to understand how participants in BWL programs use these tools. A recent systematic review analyzed randomized controlled trials of BWL interventions that incorporated digital SM (at least 12 weeks of treatment and 6-month outcome assessments) and found that 48% (23/48) of studies prescribed daily SM of weight, 69% (37/54) prescribed daily SM of diet, and 71% (39/55) prescribed daily SM of exercise. Across the intervention periods (median 6, range 3-24 months), 58% of studies achieved rates of ≥50% for SM engagement, and only 9% reached ≥75% engagement [7]. SM rates decreased over time, with only 45% of the studies showing SM rates of ≥50% by 6 months (n=33), and 38% had rates of ≥50% by ≥12 months (n=8). Engagement was the highest for SM of weight, followed by SM of diet and exercise. Synthesis of these results suggests that, although higher than rates of engagement for traditional SM methods, rates of engagement with digital SM tools are modest, and difficulties with sustaining adherence over time remain prevalent. Within this body of work, however, few prior studies have included assessment beyond 6 months; therefore, the dynamics of digital SM during the WLM phase are still unclear. Further work is needed to conceptualize digital SM engagement during this critical period (eg, 6 months and beyond when SM rates decline).

To explore this question, this project is focused on SM behavior during the WLM phase of a previously published clinical trial that assessed whether providing weight loss coaches with access to participants’ digital SM data enhances outcomes during lifestyle modification (LM) [11]. A previous publication from this parent study found that participants enrolled in a WLM intervention were more likely to self-monitor weight and eating behavior when coaches remotely monitored their data and used the data to drive treatment contacts (SMS text messages and telephone calls) versus when coaches did not have access to their data. Data sharing was also associated with less weight regain over time (although total weight loss did not differ by condition), and the frequency of dietary SM mediated the effect of treatment condition on weight loss [11]. This prior report supports the central role of SM for weight loss and maintenance and suggests that providing coaches with access to data via digital SM tools may maximize the efficacy of these tools for long-term weight control. Nevertheless, no previous analyses have been conducted to understand the nuanced patterns of SM behavior during the WLM phase when participant adherence becomes more variable owing to the difficulty of sustaining behavior change over long periods.

Furthermore, the majority of prior work on digital SM tools attempts to understand their use through the lens of percentage of adherence based on prescribed frequency, with most studies reporting the mean percentage of days that participants successfully self-monitor or the percentage of participants who maintained a certain level of SM [7]. A more nuanced exploration of SM would be helpful, including the timing of *disengagement* and rates of complete disengagement (ie, 0 days tracked) as well as how patterns of engagement vary across participants; for example, Robertson et al [12] used profile analyses to assess different patterns of SM engagement. The results showed 4 distinct profiles of use for digital SM tools among participants enrolled in a 6-month workplace weight loss intervention: minimal users (29% of the sample), activity trackers (55%), dedicated all-around users (11%), and dedicated all-around users with exceptional food logging (5%) [12]. Weight outcomes were only substantially better among the dedicated all-around users with exceptional food logging, aligning with other work that highlights the importance of dietary SM for weight loss [9,13]. Another study focused on self-weighing behavior during a 12-month BWL treatment and found 3 profiles: high/consistent (75% of the sample; SM of weight >6 days per week regularly), moderate/declined (16.2%; SM of weight 4-5 days per week, then declined to 2 days gradually) and minimal/declined (8.8%; SM of weight 5-6 days per week, then declined to 0 days suddenly), with the high/consistent group losing more weight at 6 and 12 months [14]. These findings show intriguing preliminary evidence for between-persons variation in patterns of engagement with digital SM tools, which has implications for weight loss success and needs further research, particularly during the WLM period.

Another gap in the literature on digital SM tools for weight control surrounds predictors and moderators of engagement. Little is known about what individual-level variables relate to strong adherence to SM prescriptions in BWL programs. Some previous work has found that higher initial weight loss [15], enhanced social support [16,17], and heightened binge eating severity [18] were associated with higher rates of SM during BWL programs (uncontrolled eating and emotional eating were explored as predictors of SM engagement but were not substantially related) [18]. There is also theoretical support for the idea that previous SM behavior is likely a strong predictor of consistent long-term use of SM, given that past behavior is a strong indicator of future behavior [14,19]. To our knowledge, no studies have explored individual-level predictors of long-term use of digital SM tools during WLM.

Weight-related information avoidance, which is the tendency to prevent or delay acquisition of potentially unwanted weight-related information [20], has strong theoretical support for a relationship with digital SM use. Digital SM tools provide BWL program participants immediate detailed information on progress and goal attainment, which should increase awareness of current eating or exercise patterns [16,21]. This may be differentially helpful (vs distressing) for SM engagement based on an individual’s level of weight-related information avoidance. Those with low weight-related information avoidance may be eager to engage with digital SM data and find value in reflecting on patterns of behavior [22]. However, for those with high weight-related information avoidance, viewing SM data may be distressing and reduce willingness to engage in future SM [23]. Research on health information avoidance suggests that people avoid health information for three reasons as follows: (1) it may cause unpleasant emotions (ie, guilt and shame), (2) it may dictate undesired action (ie, seeing weight gain on the scale dictates a reduction in calories and change in eating habits), and (3) it may dictate a change in beliefs (ie, seeing the calories associated with one’s favorite menu item at a restaurant may dictate changing beliefs about the feasibility of incorporating it into a weight loss diet) [20]. For all these reasons, the data provided via digital SM tools have the potential to be highly upsetting for those with high health information avoidance. This can have long-term implications for SM engagement because avoidance is likely to continue owing to negative reinforcement (ie, avoidance decreases distress associated with confronting weight-related information). Previous work from the initial BWL phase of the parent study for this analysis shows a relationship between higher weight-related information avoidance and poorer SM of exercise and weight but not diet [24]. Other studies have found that confronting information that can be perceived as a failure (eg, high calorie intake and weight gain) is associated with a higher likelihood of avoiding subsequent SM (eg, self-weighing) [25,26], supporting a relationship between health information avoidance and SM engagement. Further work is needed to replicate these results and explore the relationships during WLM to see whether health information avoidance continues to predict decreased SM engagement as time progresses.

There is also a theoretical and empirical rationale for a relationship between weight bias internalization and digital SM engagement during WLM. Weight bias internalization happens when individuals are aware of negative stereotypes associated with weight, apply these stereotypes to themselves, and engage in self-critical dialogue because of their body size [27]. This negative self-concept (eg, being lazy and lacking willpower) can be associated with lower confidence and self-efficacy [28], which is a consistent predictor of poorer engagement in weight control behaviors during LM [29] and lower weight loss success [30,31]. As participants view data from their digital SM tools and reflect on progress, the lack of goal attainment may contribute to feelings of failure or frustration. For those with high internalized weight bias, these perceived failures may be associated with more intense experiences of shame, guilt, and self-blame than for those with low internalized weight bias, further worsening their confidence and self-efficacy and leading to decreased engagement in SM [28,32]. In addition, in some cases (including the parent study), digital SM information is addressed by BWL coaches who monitor participant progress and provide personalized feedback (eg, SMS text messages) based on data [11]. Although this is meant to enhance supportive accountability, allow coaches to provide more tailored feedback, and increase motivation, this type of surveillance may deter those with high weight bias internalization from engaging in digital SM because they may have heightened sensitivity to the shame surrounding potential negative evaluations that may occur while others are monitoring their data [33,34]. A small body of prior work shows a relationship between high internalized weight bias and lower rates of SM engagement among those attempting weight loss [27,35], but this has not been examined in WLM.

### Objectives

In line with precision medicine initiatives [36], insight into individual characteristics (weight-related information avoidance and weight bias internalization) that influence SM engagement will help to drive more tailored intervention approaches for those who may be at risk for poor adherence to this key weight control behavior. This study aimed to address current gaps in the literature by exploring patterns of adherence to daily SM of weight, diet, and exercise via digital tools during the WLM phase of a BWL program (aim 1). Among BWL program participants with low SM adherence, this study also examined timing and patterns of disengagement and explored the extent to which participants reengaged with SM after low rates of earlier engagement (aim 2). Finally, this study also sought to determine how individual-level factors (weight-related information avoidance and weight bias internalization) were associated with SM of weight, exercise, and diet during WLM (aim 3).

## Methods

### Overview

This study is a secondary analysis of data from a completed randomized controlled trial (ClinicalTrials.gov NCT03337139) [11] assessing whether coach contact with access to digital SM data enhanced WLM outcomes compared with coach contact without access to digital SM data. Participants (N=77) were adults (aged 18-70 years) with overweight or obesity (BMI 25-45 kg/m^2^) who had access to a smartphone and internet and could safely engage in exercise. The exclusion criteria of the parent study included a medical or psychiatric condition that posed a risk for program adherence or safety; pregnancy or plan to become pregnant or move from study area; the use of pacemaker; a history of bariatric surgery; recent start of, or change to, a medication that can affect weight; and weight loss of ≥10% in the past 3 months.

### Ethics Approval and Informed Consent

The parent study was approved by the Drexel University Institutional Review Board (institutional review board protocol number: 1611004954), and all participants provided written informed consent before participation.

### Study Flow and Description of BWL Treatment

**Figure 1:**
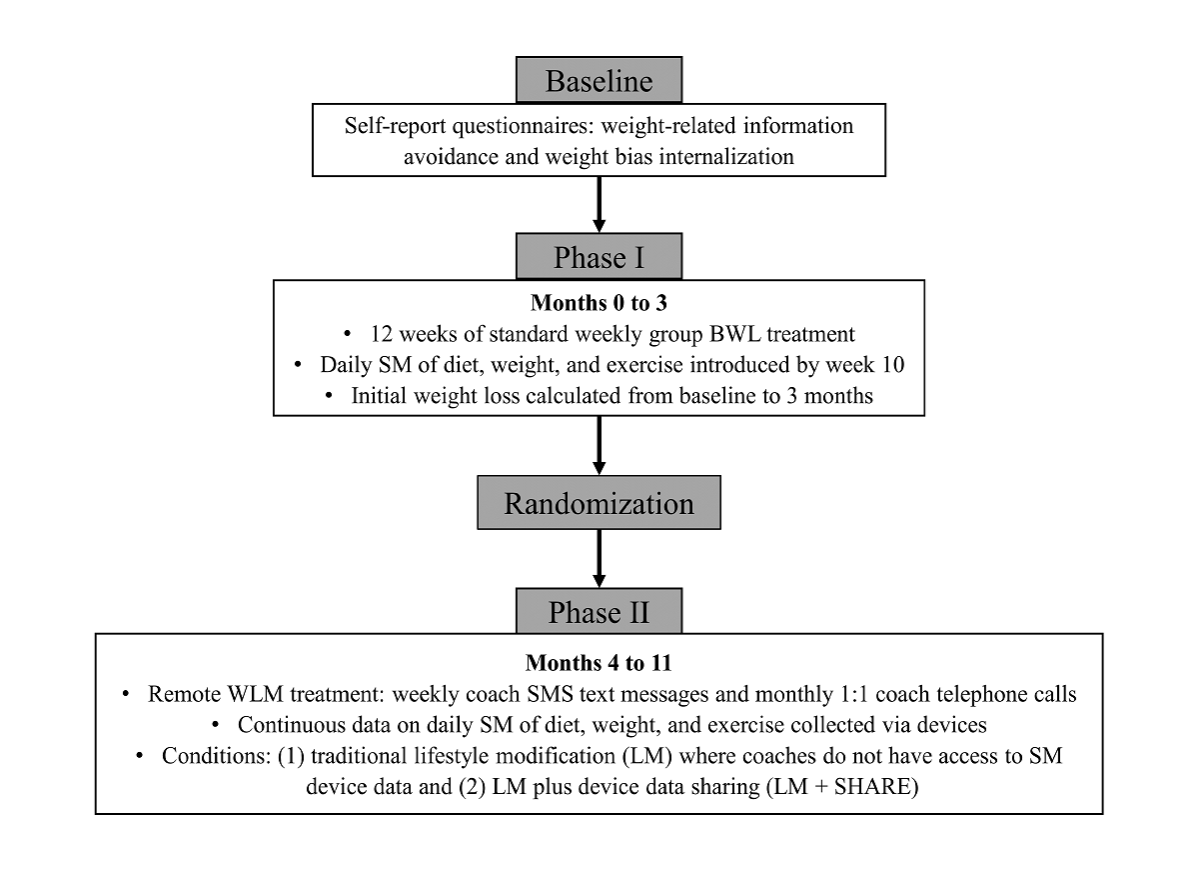
Summary of study flow, including data collection and treatment details. BWL: behavioral weight loss; LM: lifestyle modification; LM+SHARE: lifestyle modification plus device data sharing; SM: self-monitoring.

For a summary of the flow of the study, refer to Figure 1. During months 0 to 3 (phase I), all participants received 12 weeks of standard weekly group BWL treatment, which included tailored calorie goals, traditional behavioral skills adapted from the Look AHEAD (Action for Health in Diabetes) and Diabetes Prevention programs [37,38] (eg, goal setting and problem-solving), and progressive exercise goals of up to 250 minutes per week. Coaches had training in BWL treatment and degrees in psychology or a related field. All participants were provided with digital tools to track weight, exercise, and diet: Yunmai smart scale (weight), Fitbit Flex (exercise; Fitbit Inc), and Fitbit app (diet). Participants were instructed to (1) weigh themselves weekly during weeks 1 to 10 and then weigh daily, (2) wear their Fitbit Flex daily to monitor active minutes, and (3) log all food and drink intake daily. Coaches did not have access to device data during phase I.

At the end of phase I, participants were randomly assigned (matched for phase I weight loss) to 1 of 2 remote WLM treatment conditions for months 4 to 11. Both conditions included weekly SMS text messages with a coach and monthly one-on-one coach telephone calls (15 minutes) that reviewed ≥1 core behavioral skills taught during group sessions. All participants were prescribed continued daily SM of weight, diet, and exercise throughout phase II. Monthly coach calls were focused on positive reinforcement and self-reflection when participants were succeeding with behavior changes and weight loss goals and focused on problem-solving barriers or fostering motivation when participants were struggling with goal attainment. The conditions differed in terms of coaches’ access to participants’ digital SM data. In the standard LM condition, coaches did not have access to SM device data. Instead, participants self-reported goal progress during monthly calls, and coaches used that self-report to drive discussion of behavioral skills. In the LM condition, weekly SMS text messages were standardized across participants and were not personalized by a coach. In the LM plus device data sharing (LM+SHARE) condition, coaches viewed participants’ SM outcomes on a web-based portal and used these data to personalize telephone calls and SMS text messages. Coaches were trained in how to use data to enhance a sense of supportive accountability and to use data to drive more tailored personalized feedback and goal setting. SM adherence was a key discussion point during monthly coach telephone calls, given the critical role of SM behavior for WLM. Whether coaches were viewing participants’ SM data themselves (LM+SHARE condition) or reacting to participants’ self-reported SM adherence (LM condition), they were trained to identify barriers to success, facilitate effective problem-solving and tailored goal setting, and engage in motivational enhancement when adherence was poor. Coaches handled the lack of goal achievement and lack of weight loss progress in similar ways. The results from the parent study indicated that participants in the LM+SHARE condition had higher rates of weight and dietary SM [11]; therefore, treatment condition will be controlled for in analyses.

### Measures

#### Data Collection

Assessments were completed at baseline, month 3 (the end of phase I and beginning of phase II), month 6, and month 12 (the end of phase II). This study used self-report questionnaire data from baseline assessments as well as continuous SM data collected daily from participants’ devices throughout phase II (months 4-11). The participants’ SM devices (Fitbit Flex, wireless scale, and Fitbit dietary SM app) automatically uploaded data remotely to a research portal. Thus, once SM data were recorded by the participants’ SM devices, there was no additional burden on participants to transfer these data to the research team.

#### Weight-Related Information Avoidance

An adapted version of the Information Avoidance Scale (IAS) [39] was created for the parent study to assess the level of weight-related information avoidance surrounding key weight control behaviors. The 10 items on this self-report measure include statements about attitudes or tendencies to seek out versus avoid information about calorie intake, physical activity, and weight. At baseline, participants responded to each statement on a 7-point Likert scale (ranging from 1=*strongly disagree* to 7=*strongly agree*). Total scores were calculated as the average across the 10 items. The measure created showed strong internal consistency (Cronbach α=.85) [24].

#### Weight Bias Internalization

The Weight Bias Internalization Scale (WBIS) [40] is an 11-item self-report measure that assesses the extent to which the respondent believes that negative stereotypes or self-statements about weight apply to them. At baseline only, participants were presented with certain statements (eg, “As an overweight person, I feel that I am just as competent as anyone”) and asked to rate their agreement on a 7-point Likert scale (ranging from 1=*strongly disagree* to 7=*strongly agree*). Total scores are calculated as the average rating across the 11 items. The questionnaire has high internal consistency and construct validity [40].

#### SM Adherence (Phase I and Phase II)

For all months 1 to 11, the percentage of days per month that participants successfully self-monitored weight, diet, and exercise was calculated to create an average monthly adherence score for each month. For each participant, average overall phase I (months 1-3) and phase II (months 4-11) adherence scores were also calculated for each SM target (separate variables for phase I vs phase II). A valid day of exercise SM was defined as logging ≥500 steps, and a valid day of dietary SM was defined as logging ≥5 foods, both of which have precedent in the literature [24,41,42].

#### Patterns of SM Engagement and Adherence

Several metrics were calculated to understand patterns of SM engagement throughout phase II (calculated separately for SM of exercise, weight, and diet). A cutoff of 50% of days was chosen as a threshold to define *low* versus *high* adherence to SM and was selected for several reasons. First, previous work has used 50% as a cutoff for defining SM adherence versus nonadherence [43], and moderate adherence has been defined as 12 to 16 days per month (approximately 50%) [14]. In addition, the systematic review of digital SM within BWL interventions showed that, by months 6 to 12 of the intervention period, engagement rates of ≥50% were achieved in only a minority of studies (38%-45%) [7], suggesting that adherence of >50% is relatively difficult to achieve. Finally, within this sample, exploratory analyses were conducted to ensure that a 50% cutoff indicated a meaningful shift in adherence rates rather than adherence hovering right around 50% (eg, changing from 52% to 48%), which would not necessarily be clinically relevant. For each participant, the first month in which average adherence dropped to <50% (separate for each SM target) was identified as their drop-off month. For SM of weight, diet, and exercise, 2-tailed paired sample *t* tests confirmed that average monthly adherence in the month before drop-off was significantly higher than adherence during the drop-off month, and average monthly adherence in the month after drop-off was significantly lower. This further supports the use of 50% as a clinically relevant metric for high versus low adherence because adherence meaningfully shifts before and after reaching this cutoff.

The percentage of participants who maintained high adherence (≥50%) throughout all of phase II was calculated, as well as the typical time during phase II where low adherence first occurs (ie, early in WLM during months 4-7 or late in WLM during months 8-11). For those participants whose adherence dropped to <50%, rates of reengagement were also calculated by establishing the number of participants who successfully rebounded to adherence rates of ≥50% at some point during the rest of phase II. The total number of months that participants exhibited complete disengagement (0% adherence) and the number of consecutive months that participants completely disengaged during phase II were also calculated for each SM target. Adherence to SM during phase II was also compared with participants’ phase I SM adherence. The month in which adherence for each SM target dropped by ≥10% compared with average adherence during phase I was identified, as well as the number of months that participants maintained adherence equal to the average of phase I. In addition, we investigated whether participants’ adherence successfully rebounded back to phase I levels once it dropped in phase II.

### Data Analysis

All data analyses were conducted in SPSS (version 28; IBM Corp) and SAS (version 9.4; SAS Institute Inc) software, and α was set to .05. All data were screened before statistical testing to assess for outliers and normality. The distributions for weight-related information avoidance scores and for SM adherence during phase II were nonnormally distributed. Although most parametric tests are robust to skewness [44], nonparametric tests and bootstrapping were conducted for analyses using these variables as sensitivity analyses. Given the results from the parent study showing differences in weight and dietary SM between the LM and LM+SHARE conditions [11], our results are reported separately by condition where appropriate to help illustrate any differences between the groups.

For *aim 1*, descriptive statistics for adherence to SM of weight, diet, and exercise throughout phase II were calculated, within each month and across all of phase II (months 4-11). The percentages of participants who maintained high adherence and those who maintained low adherence were also calculated. Repeated-measures ANOVA (robust to assumptions of nonnormality [45]) assessed for differences in average phase II adherence rates across SM of diet versus weight versus exercise. Multilevel modeling was used to examine changes in adherence rates for each type of SM time in phase II (ie, month in study; level 1) while accounting for between-person variance (level 2). Analyses also controlled for study condition (level 2), and the time × condition interaction was explored too. Chi-square likelihood tests examined whether the inclusion of random participant slope effects improved model fit. The results of the best-fitting model are presented.

For *aim 2*, all aforementioned SM engagement variables (eg, month adherence dropped to <50% and month adherence dropped to <phase I average adherence) were calculated for each SM target, and descriptive statistics were calculated. Repeated-measures ANOVA assessed for differences in average month where adherence dropped to <50% and <phase I average across SM of diet, weight, and exercise (separate models).

For *aim 3*, Pearson correlations were used to assess relationships between phase II SM adherence for weight, diet, and exercise and weight-related information avoidance and weight bias internalization (bootstrapping with 1000 samples was conducted as sensitivity analysis to confirm results in nonnormally distributed variables). Two-tailed independent samples *t* tests (Mann-Whitney *U* tests for nonnormally distributed variables) were used to assess group differences in weight-related information avoidance and weight bias internalization between participants with low phase II adherence and those with high phase II adherence on each SM target. Using iterative multilevel model building procedures, we also tested cross-level interactions between the hypothesized person-level predictors (ie, weight bias internalization and weight-related information avoidance; level 2) and time (ie, month in study; level 1) for each type of SM adherence. Between-person variables were grand-mean centered. Cross-level interactions were compared with random slope models for fit. For significant cross-level interactions, simple slopes were calculated and graphed to depict SM adherence across time at the mean of, as well as 1 SD above and 1 SD below, the between-person predictor, which allows for better visualization of interaction effects and in a way that is more interpretable and clinically meaningful [44].

## Results

### Descriptive Statistics

Of the 77 participants, 72 (94%) provided phase II data and were included in these analyses. Participants were on average aged 51.27 (SD 13.47) years, predominantly female (58/72, 81%), and non-Hispanic/Latino (69/72, 96%). Approximately half of the participants (37/72, 51%) identified as White, 38% (27/72) as Black/African American, 7% (5/72) as other or >1 race, 3% (2/72) as Asian, and 1% (1/72) as American Indian/Alaska Native. On average, participants lost 5.89% (SD 4.31%) of their body weight during phase I. Higher percentage of weight loss during phase I was correlated with higher engagement in SM of weight (*r*=−0.28; *P*=.02), diet (*r*=−0.41; *P*<.001), and exercise (*r*=−0.26; *P*=.03) in phase I. The relationships between previous SM behavior (during phase I) and SM engagement during phase II can be seen in Table 1.

**Table 1:** The correlation matrix of individual-level variables and phase II adherence for each self-monitoring (SM) target.

Variable, mean (SD)			Average adherence to SM of weight in phase II		Average adherence to SM of diet in phase II		Average adherence to SM of exercise in phase II		Baseline IAS^a^ Score		Baseline WBIS^b^ Score		Average adherence to SM of weight in phase I		Average adherence to SM of diet in phase I		Average adherence to SM of exercise in phase I
**Average adherence to SM of weight in phase II, 53.2% (3%)**																	
	*r*	—^c^		—		—		—		—		—		—		—	
	*P* value	—		—		—		—		—		—		—		—	
**Average adherence to SM of diet in phase II, 49.34% (2.9%)**																	
	*r*	*0.638* ^d^		—		—		—		—		—		—		—	
	*P* value	*<.001*		—		—		—		—		—		—		—	
**Average adherence to SM of exercise in phase II, 80.01% (2.3%)**																	
	*r*	*0.581*		*0.604*		—		—		—		—		—		—	
	*P* value	*<.001*		*<.001*		—		—		—		—		—		—	
**Baseline IAS Score, 2.13 (0.96)**																	
	*r*	0.048		−0.108		−0.067		—		—		—		—		—	
	*P* value	.68		.36		.57		—		—		—		—		—	
**Baseline WBIS Score, 3.58 (1.09)**																	
	*r*	0.001		−0.085		0.073		0.228		—		—		—		—	
	*P* value	.99		.48		.55		.06		—		—		—		—	
**Average adherence to SM of weight in phase I, 88.25% (1.5%)**																	
	*r*	*0.443*		*0.257*		*0.381*		−*0.240*		0.094		—		—		—	
	*P* value	*<.001*		*.03*		*<.001*		*.04*		.44		—		—		—	
**Average adherence to SM of diet in phase I, 86.63% (1.5%)**																	
	*r*	*0.337*		*0.543*		*0.592*		−*0.203*		0.103		−*0.537*		—		—	
	*P* value	*.004*		*<.001*		*<.001*		*.09*		.40		*<.001*		—		—	
**Average adherence to SM of exercise in phase I, 94.13% (1.4%)**																	
	*r*	*0.320*		0.213		*0.421*		−*0.316*		−0.026		−*0.765*		−*0.587*		—	
	*P* value	*.006*		.07		*<.001*		*.007*		.83		*<.001*		*<.001*		—	

^a^IAS: Information Avoidance Scale.

^b^WBIS: Weight Bias Internalization Scale.

^c^Not applicable.

^d^Italics denotes significance (meeting threshold of *P*<.05).

### Aim 1

**Figure 2:**
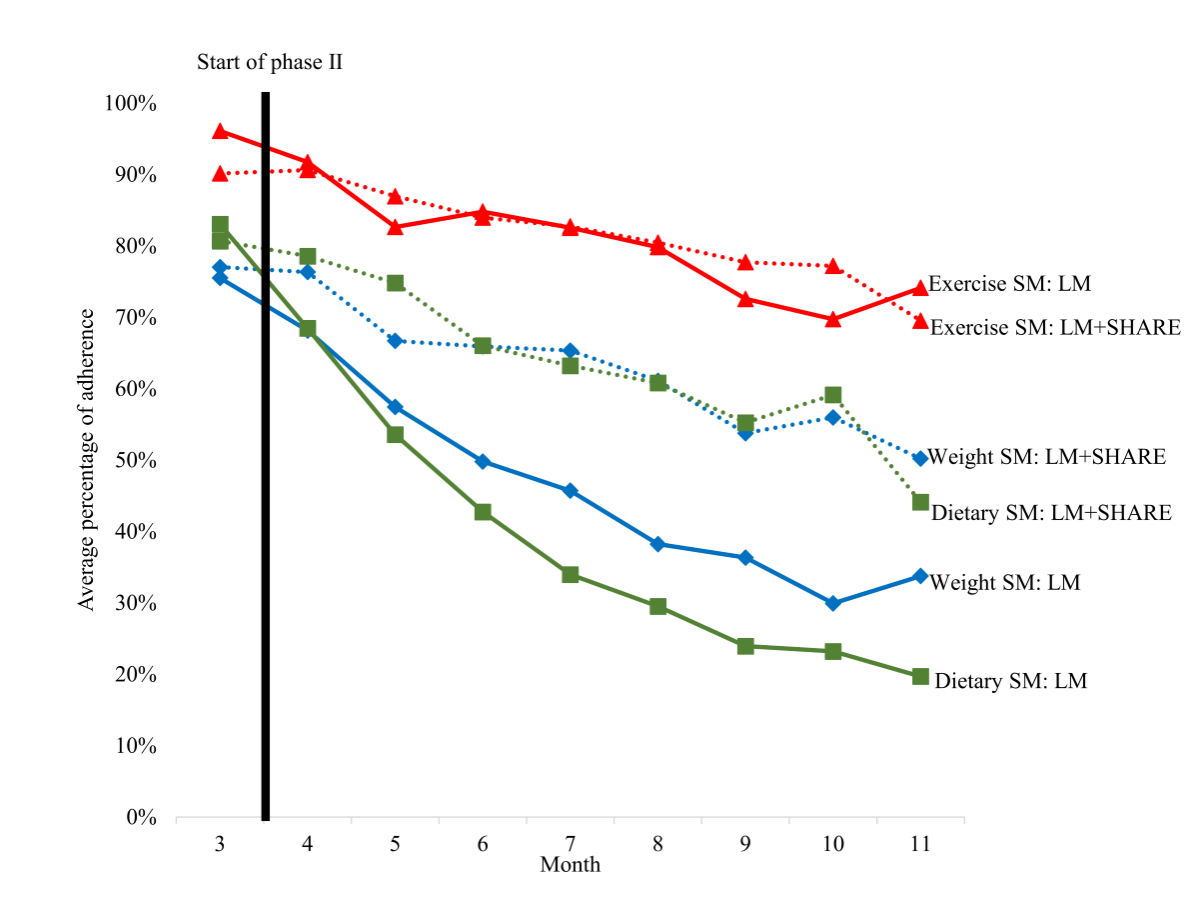
Average percentage adherence by month for self-monitoring (SM) of weight, diet, and exercise separated by condition (lifestyle modification [LM] vs LM plus device data sharing [LM+SHARE]); this includes SM adherence at the end of phase I (month 3) and throughout phase II (months 4-11).

At the end of phase I (month 3), 86% (62/72) of the participants had high adherence (≥50%) to SM of weight, 88% (63/72) had high adherence to SM of diet, and 97% (70/72) had high adherence to SM of exercise, indicating that most of the participants were still actively engaged with SM at the end of month 3 and presumably entered phase II with goals to maintain that behavior. During the WLM phase, consistently high rates (≥50% for every month) of SM were observed for 61% (44/72) of the participants for exercise, 40% (29/72) of the participants for weight, and 21% (15/72) of the participants for diet. Throughout phase II, the average percentage of adherence for SM of exercise (mean 80.01%, SD 2.3%) was significantly higher than the percentage of adherence to self-weighing (mean 53.2%, SD 3%) or food logging (mean 49.34%, SD 2.9%; *F*_2,142_=64.95; *P*<.001; η^2^_p_=0.48). Adherence to SM of weight and diet were not significantly different (*P*=.63). Only 13% (9/72) of the participants consistently exhibited ≥50% adherence on all 3 SM targets. The best-fitting multilevel models examining the effect of time on all types of SM adherence retained a random slope. The average rates of SM adherence for weight (*b*=−0.05, SE 0.01; t_71_=−8.75; *P*<.001), diet (*b*=−0.06, SE 0.00; t_71_=−12.30; *P*<.001), and exercise (*b*=−0.03, SE 0.00; t_71_=−6.53; *P*<.001) significantly decreased over time throughout phase II (months 4-11) when controlling for study condition. Refer to Figure 2 for a depiction of monthly adherence for each type of SM across time for the LM versus LM+SHARE conditions. The models tested a significant time × condition interaction, but none of the interactions were significant (weight SM: *P*=.16; dietary SM: *P*=.13; and exercise SM: *P*=.71).

### Aim 2

#### Average Time to <50% Adherence by SM Type

Average adherence dropped to <50% at 10.07 (SD 2.83) months for SM of exercise, at 7.92 (SD 3.23) months for SM of weight, and at 7.58 (SD 2.92) months for SM of diet. The average month of drop-off for adherence to SM of exercise was significantly later in WLM than drop-off for adherence to SM of weight or diet (*F*_2,142_=33.22; *P*<.001; η^2^_p_=0.32), but they did not differ from each other (*P*=.97). In the LM condition, average adherence dropped to <50% at 9.86 (SD 2.92) months for SM of exercise, at 7.03 (SD 2.95) months for SM of weight, and at 6.31 (SD 2.36) months for SM of diet. In the LM+SHARE condition, average adherence dropped to <50% at 10.27 (SD 2.76) months for SM of exercise, at 8.76 (SD 3.30) months for SM of weight, and at 8.78 (SD 2.92) months for SM of diet. Among those with low engagement at some point during phase II, the majority disengaged with SM early (months 4-7) rather than late (42/57, 74%, disengaged early for SM of diet; 35/51, 69%, disengaged early for SM of weight; and 16/28, 57%, disengaged early for SM of exercise).

#### Average Time to Drop of ≥10% From Original Adherence by SM Type

When comparing participants’ SM adherence during phase II to their phase I average adherence, adherence dropped by ≥10% compared with phase I adherence at 7.97 (SD 3.16) months for SM of exercise, at 5.88 (SD 2.63) months for SM of weight, and at 5.82 (SD 2.52) months for SM of diet. This occurred significantly later for SM of exercise than for SM of diet or weight (*F*_2,142_=19.53; *P*<.001; η^2^_p_=0.22). Throughout WLM, participants achieved rates of adherence that were at, or above, their phase I average during more months for SM of exercise (mean 5.35, SD 2.49 months) than for SM of weight (mean 2.42, SD 2.69 months) and diet (mean 2.35, SD 2.56 months; *F*_2,142_=56.73; *P*<.001; η^2^_p_=0.44).

#### Reengagement

Analyses examined the likelihood of participants returning to high adherence (ie, ≥50% of the days in any month) after a period of low adherence (ie, <50% of the days in a month). For SM of exercise, 46% (13/28) of the participants rebounded back to high adherence, whereas only one-third of the participants rebounded for SM of weight or diet (17/51, 33%, for weight and 19/57, 33%, for diet). Among those who successfully reengaged with SM of weight, the first month of rebounded rates of ≥50% tended to be month 7.94 (SD 2.05) compared with month 8.16 (SD 1.80) for SM of diet and month 7.15 (SD 2.19) for SM of exercise. When rates of adherence fell by ≥10% below the phase I average, only 30% (19/64) of the participants went on to achieve weight SM adherence rates at or above phase I levels compared with 29% (19/65) of the participants for dietary SM and 69% (36/52) of the participants for exercise SM. When rates dropped below the phase I average, those who reengaged tended to do so at 7.53 (SD 1.84) months for weight SM, at 6.53 (SD 1.65) months for dietary SM, and at 7.56 (SD 1.86) months for exercise SM.

#### Patterns of Complete Disengagement (0% Adherence)

When looking at complete disengagement (0% monthly adherence), 43% (31/72) of the participants had at least 1 full month of complete disengagement from SM of diet, and 32% (23/72) had at least 1 full month with complete disengagement from SM of weight, whereas only 19% (14/72) totally disengaged from SM of exercise for a full month. Participants who completely disengaged from self-weighing did so for an average of 2.96 (SD 1.52) months. Those who completely disengaged from SM of diet did so for an average of 3.42 (SD 2.08) months, and those who disengaged from SM of exercise did so for an average of 3.00 (SD 1.66) months. For all 3 SM targets, the months of total disengagement tended to occur consecutively for most of the participants (15/31, 48% to 9/14, 64%), rather than as a pattern where adherence increased and then decreased back down to zero.

### Aim 3

#### Weight SM

The patterns of the Pearson correlation analyses with bootstrapping and those without bootstrapping remained the same; therefore, for ease of interpretation, the results of Pearson correlations without bootstrapping are reported (Table 1). Phase II SM of weight was significantly correlated with past SM behavior during phase I (weight: *r*=0.44; *P*<.001; diet: *r*=0.34; *P*=.004; and exercise: *r*=0.32; *P*=.006) such that participants who had higher engagement on any of the SM targets throughout phase I engaged in more self-weighing in phase II. The average adherence to SM of weight throughout phase II was not correlated with baseline weight-related information avoidance or weight bias internalization.

When dichotomizing the sample into 2 groups based on self-weighing adherence (those who maintained high adherence to weight SM throughout all of months 4-11 and those who did not; Table 2), individuals who maintained consistently high self-weighing adherence in months 4 to 11 had higher baseline weight bias internalization scores than those who did not maintain high adherence to self-weighing (*U*=700.50*; P*=.03).

**Table 2:** Results of group comparisons (high vs low self-monitoring [SM] adherence) on baseline levels of weight-related information avoidance and weight bias internalization.

	Weight SM		Dietary SM										Exercise SM							
	High adherence^a^, median^b^	Low adherence, median	High adherence				Low adherence					High adherence						Low adherence		
			Median	Mean (SD)^c^			Median		Mean (SD)		Median				Mean (SD)		Median			Mean (SD)
Baseline IAS^d^ score	2.11	1.80	1.80		N/A^e^	2.10		N/A		2.10				N/A		1.80			N/A	
Baseline WBIS^f^ score	*4.27* ^g^	3.36	N/A		3.42 (1.21)	N/A		3.62 (1.07)		N/A				3.75 (1.18)		N/A			3.30 (0.89)	

^a^The high-adherence group maintained rates of ≥50% throughout all of months 4 to 11.

^b^Mann-Whitney *U* tests were used for nonnormally distributed variables, and medians are reported (due to skewness).

^c^ Two-tailed independent samples *t* tests were used for normally distributed variables and mean (SD) values are reported.

^d^IAS: Information Avoidance Scale.

^e^N/A: not applicable.

^f^WBIS: Weight Bias Internalization Scale.

^g^*P*=.03 (significant difference from low-adherence group).

Cross-level interaction models examined whether baseline weight-related information avoidance and weight bias internalization moderated the influence of time on weight SM. Cross-level interaction models were not significant (*P*=.16 and *P*=.29, respectively) and did not improve model fit compared with the random slope models examining the influence of time on weight SM tested in aim 1.

#### Dietary SM

As seen in Table 1, average phase II adherence for SM of diet was unrelated to baseline weight-related information avoidance and weight bias internalization scores. Phase II dietary SM was significantly correlated with phase I SM of weight (*r*=0.26; *P*=.03) and diet (*r*=0.54; *P*<.001) but not exercise *(r*=0.21; *P*=.07). Participants who had higher engagement with dietary and weight SM previously tended to engage in more food logging during phase II. Group comparisons between those who maintained high adherence to dietary SM during months 4 to 11 and those who did not can be seen in Table 2. The groups did not differ on baseline weight-related information avoidance or weight bias internalization (none of the *P* values met the threshold for statistical significance).

**Figure 3:**
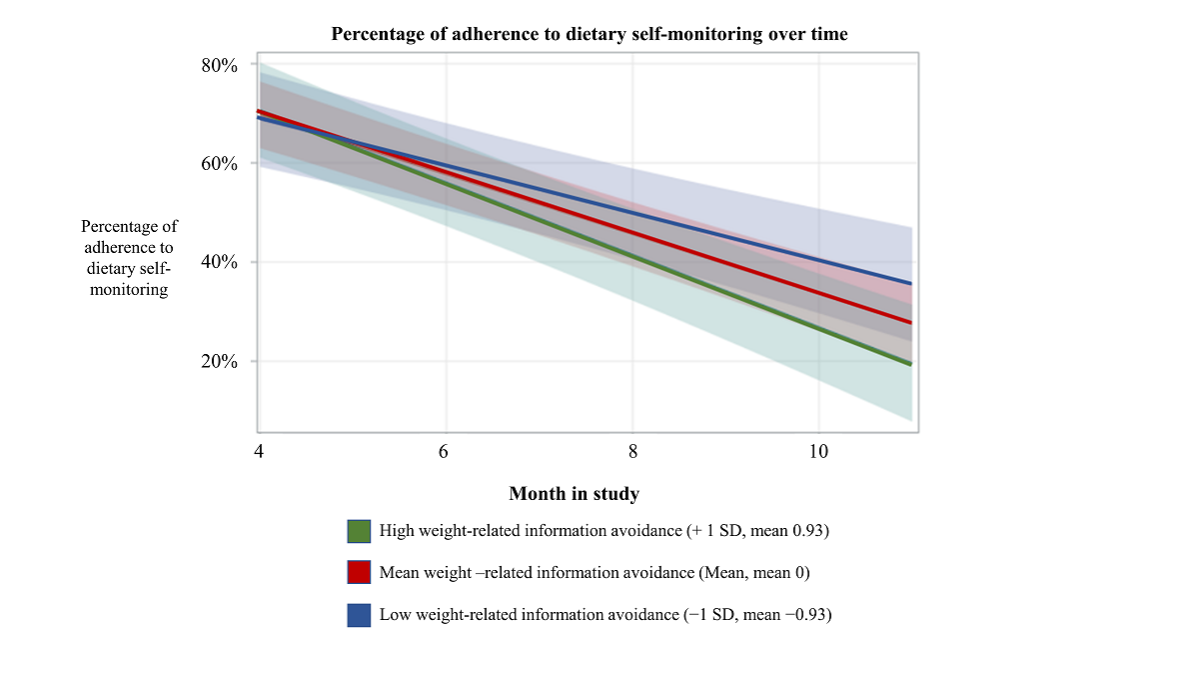
Visualization of the cross-level interaction between weight-related information avoidance and time on dietary self-monitoring adherence (weight-related information avoidance was centered within person; more positive scores indicate more avoidance).

Cross-level interaction models examined whether baseline weight-related information avoidance and weight bias internalization moderated the influence of time on dietary SM. There was a significant interaction between weight-related information avoidance and time on dietary SM (*b*=−0.01, SE 0.01; t_73.7_=−2.13; *P*=.04). The cross-level interaction model fit was significantly improved from the random slope model of time on dietary SM tested in aim 1. Simple slope analyses found that participants with high (*b*=−0.07, SE 0.01; t_74.4_=−8.61; *P*<.001) and moderate weight-related information avoidance (*b*=−0.06, SE 0.01; t_69.8_=−10.38; *P*<.001) had a steeper decline in dietary SM over time than those with low weight-related information avoidance (*b*=−0.05, SE 0.01; t_69.0_=−5.38; *P*<.001). Refer to Figure 3 for a depiction of this interaction. The cross-level interaction between weight bias internalization and time on dietary SM was not significant and did not improve model fit *P*=.08).

#### Exercise SM

Average phase II adherence for exercise SM was not correlated with baseline weight-related information avoidance or baseline weight bias internalization scores (Table 1). Average phase II adherence for exercise SM was significantly correlated with phase I SM engagement for weight (*r*=0.38; *P*<.001), diet (*r*=0.59; *P*<.001), and exercise *(r*=0.42; *P*<.001) such that participants who had higher engagement on any of the SM targets throughout phase I engaged in more consistent exercise tracking during phase II. As seen in Table 2, participants who achieved consistently high adherence to exercise SM in phase II did not differ from those with low adherence on weight bias internalization or health information avoidance.

Cross-level interaction models examined whether between-person factors moderated the effect of time on exercise SM. The cross-level interaction between weight bias internalization and time (and health information avoidance and time) on exercise SM were not significant and did not improve model fit *P*=.87 and *P*=.56, respectively).

## Discussion

### Overview of Study Objective

Daily SM of weight, diet, and physical activity is a common prescription in BWL programs [1] because this practice is highly predictive of participant success [2-4]. However, adherence to SM tends to wane over time, especially during the WLM phase [5,6]. Very few studies have examined patterns of adherence to different SM tools over long periods of time, and almost none have examined how individual differences predict different types of SM adherence. This study explored changes in SM of different tools across time, the unique timing and patterns of disengagement and reengagement, and whether theory-based individual-level factors could predict SM adherence among participants in a year-long BWL program. The findings can inform attempts to prevent disengagement with SM tools and ultimately lead to greater weight control success.

### Rates of SM Adherence

For all 3 SM targets, the rates of adherence declined across months 4 to 11. These data mirror results from previous analyses with these data during phase I (months 0-3), in which adherence to SM of weight and diet but not exercise decreased over 12 weeks [41]. SM adherence during phase II was strongly related to previous SM engagement during phase I, an expected association owing to the consistently strong association between past and current behavior [19]. Throughout phase II, the rates of SM of weight (53%, of the days) and diet (49%, of the days) were significantly lower than those of exercise (80%, of the days). These rates of SM of diet and weight are comparable with the median rates seen in past interventions, whereas the rates of SM of exercise were higher than usual [7]. Of the 72 participants, only 9 (13%) of the participants had strong (ie, ≥50%) adherence for all 3 SM targets throughout phase II, and total disengagement was common: almost half of the participants (31/72, 43%) had at least 1 month with no dietary SM, and almost one-third (23/72, 32%) had at least 1 month with no weight SM. SM engagement was modest in both conditions; however, the rates of participants achieving high SM adherence tended to be higher in the LM+SHARE condition, and more LM+SHARE participants achieved strong adherence across all 3 targets than those in the LM condition. These results parallel findings from the parent study on the potential benefit of coach surveillance of SM data [11]. The results suggest that nonadherence to SM, particularly of diet and weight, is a major problem in BWL treatment, although coach monitoring of SM data may provide 1 avenue for improvements.

The comparatively low rates of dietary SM are unsurprising because calorie tracking is a high-burden behavior that requires ample time and patience. Participants continually struggle with this behavior during BWL trials [46], and past research shows that this type of *active* SM (ie, calorie tracking) has lower engagement than *passive* SM (eg, wearing a Fitbit band) [7]. Efforts should be made to help participants track their food more easily, either by creating more user-friendly food tracking technology or by identifying tracking strategies that reduce participant burden without sacrificing effectiveness (eg, tracking only dietary lapses [47]). However, the low rates of self-weighing compared with the higher rates of exercise SM are notable, given that self-weighing, much like exercise tracking, is a low-burden behavior (ie, participants simply need to step on a scale). Therefore, the discrepancy between exercise SM and weight SM may be due to deliberate health information avoidance [48]. Weighing can be highly distressing for participants in BWL programs, and many people with overweight or obesity report avoiding the scale in fear of experiencing the negative feelings it may evoke [49]. Past studies show that people are less likely to weigh themselves when they have recently gained weight [50] or eaten more calories than usual [24], suggesting that the declining adherence for self-weighing may be a result of avoidance of the scale as eating and exercise behavior become less stringent than they were at the start of the program. The results of this study too suggest that efforts to enhance participant engagement with self-weighing may require addressing participant reactions to weight information (eg, with self-compassion training) rather than logistical efforts related to decreasing burden of the SM behavior.

### Patterns of Disengagement and Reengagement

Average adherence for diet and weight SM fell to <50% around 7 months into the program, whereas average adherence for exercise SM fell off later, 10 months into the program. Among those who did have low engagement, most dropped off fairly early (ie, months 4-7) in phase II. Among participants who had a meaningful decrease in adherence (ie, >10%) compared with their phase I frequency of SM, the drop-off tended to occur just before month 6 (ie, 2 months into phase II) for diet and weight and around month 8 for SM of exercise. These results suggest that participants are at risk for SM disengagement, particularly with diet and weight, around the 6-month mark of BWL programs; thus, this may be an optimal time for a potential intervention.

Only approximately one-third of the participants whose weight and dietary SM adherence dropped to <50% ever reengaged (ie, restored levels of SM to ≥50% by the end of the program;17/51, 33% for weight SM and 19/57, 33% for dietary SM). However, almost half of the participants (13/28, 46%) whose exercise SM adherence dropped to <50% were able to reinstate those levels later during WLM. Therefore, when participants disengage with SM of weight and diet in particular, they are highly unlikely to reengage. It seems to be more likely that participants will pick back up with SM of exercise even if they have had low levels of adherence previously, suggesting that this behavior is more resilient against prior difficulties. Both rates of disengagement and reengagement were more promising when data were shared with coaches (LM+SHARE), suggesting that remote coach monitoring may be 1 way to help protect participants against dropping the key weight control behavior of SM.

### Explaining SM Adherence and Adherence Trajectories

Although nonadherence to weight SM may be evidence of deliberate avoidance, self-reported weight-related information avoidance at baseline was not predictive of SM of weight or exercise. It is possible that the preference to deliberately avoid weight-related information predicts SM less strongly than expected because avoidance is a dynamic factor that changes owing to situational factors; for example, 1 study found that the preference to avoid weight-related information was associated with state variables (such as shame and negative mood) among adult women with overweight or obesity but not with trait variables (such as BMI, age, or past stigma regarding weight) [51]. Nevertheless, higher baseline weight-related information avoidance was associated with a steeper decline in *dietary* SM over time. Participants with a stronger tendency to avoid negative weight-related information may find it difficult to confront their calorie intake when they expect that the numbers will elicit shame [49]. As time progresses in WLM, and more participants drift from their calorie goals, recording that information seems to be more challenging for those who enter BWL treatment with higher weight-related information avoidance tendencies. Future work should confirm the dynamics of this relationship with more frequent assessment of weight-related information avoidance throughout WLM.

Contrary to expectations, in this study, baseline weight bias internalization was associated with higher adherence to weight SM. This finding is surprising because past research shows that weight bias internalization is associated with body image avoidance [52] and the avoidance of health care entirely [27]. It is possible that weight bias internalization could lead to *greater* motivation for weight loss to reduce weight-related guilt and shame; however, research consistently shows that such internalization is ultimately maladaptive [27]. Further work is needed to clarify these conflicting results and elucidate the underlying mechanism by which weight bias internalization predicts higher levels of weight SM. Research is also needed to determine whether the value of self-weighing is different between those with high weight bias internalization and those with low weight bias internalization.

### Implications for BWL Treatment

Overall, the results emphasize the fact that SM of diet and weight is a challenge during BWL treatment (even when using digital tools) and should be prioritized as intervention targets. Long-term SM adherence was associated with higher engagement at earlier points of the BWL program (phase I), suggesting that individuals who can establish a consistent, regular SM routine early in treatment will find it easier to maintain it during WLM. Deliberate weight-related information avoidance may occur, as evidenced by the low rates of SM of weight despite its being a low-burden behavior in comparison with SM of diet. Thus, strategies to increase rates of self-weighing among participants in BWL programs may be best designed to target participants’ reaction to SM (eg, self-compassion training) versus logistical problem-solving to decrease burden. This study is the first to identify potential individual-level factors related to use of digital SM tools during WLM. The findings suggest that participants in BWL programs entering treatment with higher rates of weight-related information avoidance and lower rates of weight bias internalization may be at higher risk for low long-term engagement with dietary and weight SM, respectively. This has clinical utility because these individuals can then be identified at baseline and targeted throughout treatment with specific strategies to facilitate sustaining SM as a key weight control behavior. The findings point to the utility of just-in-time adaptive interventions (JITAIs) to promote reengagement with SM tools among participants whose SM starts to decline. JITAIs are dynamic, identifying critical moments for intervention and providing tailored support [53]. Such interventions may be especially effective 6 to 8 months into treatment because, in this study, this was a critical period with high rates of disengagement from SM. Without this intervention, participants may have a difficult time resuming adherence to these crucial behaviors because the current data suggest that few participants who disengage will ever reengage. Future research should identify what psychological or practical support participants need at these times to inspire them to reengage.

### Strengths and Limitations

This study had several strengths. It included long-term assessment of the use of digital SM tools after the intensive phase of a BWL program, which was a noted gap in the literature. SM data were collected objectively from wireless scales and passive Fitbit sensors, which is a particularly valid method of data collection. Given the design of the parent study, these analyses also provided a chance to look at SM patterns with remote coach monitoring of participant SM data and those without. Data sharing with coaches is a new development within BWL treatment innovation that is not included in most interventions. Thus, it is helpful to clarify what long-term digital SM behavior looks like with coach data surveillance and without. A limitation of the study is its sample size of 77 participants, limiting power to detect small effects. Additional research in a larger, more diverse (specifically, sex diverse) sample would increase the generalizability of, and confidence in, the findings. There was also attrition throughout phase I, where 10 (11%) of the 87 participants dropped out before randomization into phase II and 5 (6%) of the 77 enrolled in phase II did not provide data for this analysis. It is possible that these individuals were more likely to have disengaged from SM and thus would have exhibited poor rates of adherence. Thus, phase II SM adherence rates may have been lower (and the results may have differed) if these analyses were conducted using a data set that included all participants. Furthermore, definitions of SM adherence (eg, high adherence: ≥50% and valid days of calorie tracking: logging ≥5 foods) were based on past literature but are still somewhat arbitrary; for example, it is unclear whether using a threshold of 800 calories per day or at least 2 eating episodes per day is a better determinant of valid calorie days [43].

### Conclusions

This study found that weight, diet, and exercise SM declined over time during the maintenance phase of a BWL intervention. Adherence to dietary SM was the poorest, followed by adherence to weight SM, whereas adherence to exercise SM was comparatively higher. Few participants maintained high levels of SM across the full study, and total disengagement from SM was common, with low rates of reengagement. These rates of adherence are particularly troubling, given the trial’s strong emphasis on SM. Higher baseline health information avoidance and lower baseline weight bias internalization were associated with poorer SM. The findings suggest that future BWL interventions may benefit from JITAIs that identify when participants are at risk for disengagement and provide adaptive support to promote better SM adherence.

## Abbreviations

AHEAD: Action for Health in Diabetes
BWL: behavioral weight loss
IAS: Information Avoidance Scale
JITAI: just-in-time adaptive intervention
LM: lifestyle modification
LM+SHARE: lifestyle modification plus device data sharing
SM: self-monitoring
WBIS: Weight Bias Internalization Scale
WLM: weight loss maintenance
